# Use of Multiple-Select Multiple-Choice Items in a Dental Undergraduate Curriculum: Retrospective Study Involving the Application of Different Scoring Methods

**DOI:** 10.2196/43792

**Published:** 2023-03-27

**Authors:** Philipp Kanzow, Dennis Schmidt, Manfred Herrmann, Torsten Wassmann, Annette Wiegand, Tobias Raupach

**Affiliations:** 1 Department of Preventive Dentistry, Periodontology and Cariology University Medical Center Göttingen Göttingen Germany; 2 Division of Medical Education Research and Curriculum Development Study Deanery of University Medical Center Göttingen Göttingen Germany; 3 Department of Prosthodontics University Medical Center Göttingen Göttingen Germany; 4 Department of Cardiology and Pneumology University Medical Center Göttingen Göttingen Germany; 5 Institute for Medical Education University Hospital Bonn Bonn Germany

**Keywords:** dental education, education system, educational assessment, educational measurement, examination, k of n, Kprim, K’, MTF, Multiple-True-False, Pick-N, scoring, scoring system, Type X, undergraduate, undergraduate curriculum, undergraduate education

## Abstract

**Background:**

Scoring and awarding credit are more complex for multiple-select items than for single-choice items. Forty-one different scoring methods were retrospectively applied to 2 multiple-select multiple-choice item types (Pick-N and Multiple-True-False [MTF]) from existing examination data.

**Objective:**

This study aimed to calculate and compare the mean scores for both item types by applying different scoring methods, and to investigate the effect of item quality on mean raw scores and the likelihood of resulting scores at or above the pass level (≥0.6).

**Methods:**

Items and responses from examinees (ie, marking events) were retrieved from previous examinations. Different scoring methods were retrospectively applied to the existing examination data to calculate corresponding examination scores. In addition, item quality was assessed using a validated checklist. Statistical analysis was performed using the Kruskal-Wallis test, Wilcoxon rank-sum test, and multiple logistic regression analysis (*P*<.05).

**Results:**

We analyzed 1931 marking events of 48 Pick-N items and 828 marking events of 18 MTF items. For both item types, scoring results widely differed between scoring methods (minimum: 0.02, maximum: 0.98; *P*<.001). Both the use of an inappropriate item type (34 items) and the presence of cues (30 items) impacted the scoring results. Inappropriately used Pick-N items resulted in lower mean raw scores (0.88 vs 0.93; *P*<.001), while inappropriately used MTF items resulted in higher mean raw scores (0.88 vs 0.85; *P*=.001). Mean raw scores were higher for MTF items with cues than for those without cues (0.91 vs 0.8; *P*<.001), while mean raw scores for Pick-N items with and without cues did not differ (0.89 vs 0.90; *P*=.09). Item quality also impacted the likelihood of resulting scores at or above the pass level (odds ratio ≤6.977).

**Conclusions:**

Educators should pay attention when using multiple-select multiple-choice items and select the most appropriate item type. Different item types, different scoring methods, and presence of cues are likely to impact examinees’ scores and overall examination results.

## Introduction

In dentistry, multiple-choice items are often used to test theoretical knowledge in written examinations [1]. Multiple-choice items can be divided into single-choice items (eg, Type A) and multiple-select items. In multiple-select items, examinees are required to judge multiple answer options/statements independently within a single item. The correctness of an answer option/statement does not affect the other answer options/statements within the same item. Therefore, a more active knowledge reproduction takes place as examinees cannot identify the correct answer option at the first glance and must not ignore the remaining answer options. In contrast to single-choice items, scoring of multiple-select items is more complex. While examinees’ responses on single-choice items might be either correct (1 full credit point is awarded) or incorrect (no credit points are awarded or a penalty score is given), multiple-select items might result in partially correct responses (ie, some answer options/statements are marked correctly while others are marked incorrectly).

**Figure 1:**
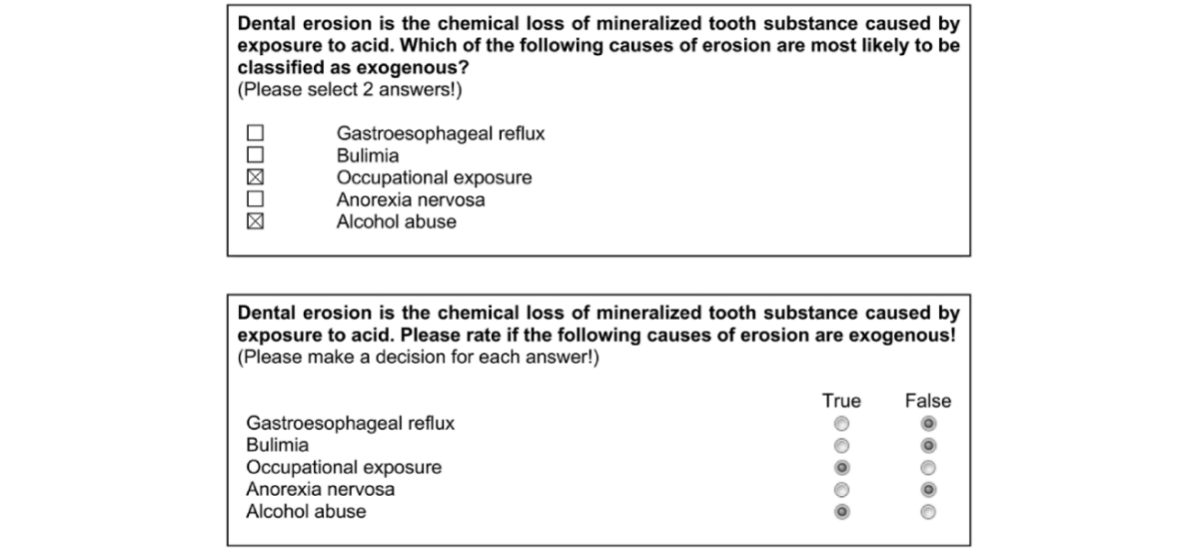
Examples of matched Pick-N (top) and Multiple-True-False (bottom) items with 5 answer options/statements.

Within electronic written examinations of dental undergraduate students at the University Medical Center Göttingen, Type A single-choice items and 2 kinds of multiple-select multiple-choice items, known as Pick-N [2,3] and Multiple-True-False (MTF) [4], are used. Examples of the item types are shown in Figure 1. Since the first mention of these item types, various scoring methods for scoring multiple-select items have been described in the literature. A summary of different scoring methods and their corresponding mathematical scoring algorithms as identified by 2 recent systematic reviews [5,6] is shown in Multimedia Appendix 1.

Pick-N items consist of a variable number of answer options (with the number [*n*] ranging from 5 to 26 [7-9]), and examinees are asked to select all true answer options. The total number of true answer options (*t*) within each item is disclosed to examinees and might vary between 2 and *n*–1 [3,7,9-11]. In recent years, Pick-N items were described to typically consist of 1 circumscript question and a number of very short answer options (ie, a single word or very short phrases) [7,10]. This item type has also been named *k from n* and *n out of many* in the literature [8,9].

MTF items consist of a question stem and a variable number of statements (ie, complex statements as opposed to very short answer options used in Pick-N items), which need to be labeled independently as true or false by examinees. Any number of statements (including zero and *n*) might be correct, and the number of true statements is not disclosed. This item type has also been named *true-false format*, *cluster-true-false*, *cluster* (*multiple true-false*) *variety*, *cluster-type true-false*, *Kprim*, *Kprime*, *K’*, and *Type X* in the literature [12-16]. Based on the above-mentioned definitions of Pick-N and MTF items, the example shown in Figure 1 should be employed as a Pick-N item instead of an MTF item.

Although indications for the use of multiple-select multiple-choice items and corresponding instructions for examinees vary between both item types [7,10], it is unknown whether educators employ Pick-N and MTF items according to the above-mentioned recommendations. Moreover, the relation between examinees’ true ability (ie, *true knowledge*) and expected scoring results differs between both item types [5,6]. In case of examinations consisting of single-choice items with 5 answer options only (ie, with a guessing probability amounting to 20%), a pass mark of 60% tests examinees for a level of 50% *true knowledge*, as examinees with 50% *true knowledge* achieve 60% of the possible total score on average due to the possibility of guessing (using an all-or-nothing scoring method without applying a penalty for incorrect responses). Depending on the employed multiple-select item type, the number of answer options/statements per item, and the used scoring method, examinees might require either more or less *true knowledge* to gain 60% of the possible total score on average.

Therefore, this study aimed to (1) retrospectively apply different scoring methods to existing examination data from multiple-select multiple-choice items and analyze the obtained results from examinees (ie, scores) and (2) investigate the impact of item characteristics (ie, selection of appropriate item type and presence of cues) on scoring results (ie, mean raw scores and the likelihood of resulting scores at or above pass level when using different scoring methods).

The null hypotheses were as follows: (1) scoring results for Pick-N and MTF items do not differ between different scoring methods and (2) item characteristics do not impact scoring results.

## Methods

### Ethical Considerations

Owing to the retrospective design of the study and the fact that only anonymized item scores at the level of previous examinations (ie, not at the level of identifiable students) were available from the examination software, no ethical approval was required.

### Multiple-Select Multiple-Choice Items

At the University Medical Center Göttingen, both Pick-N and MTF multiple-select multiple-choice items are used. While Pick-N items might contain a variable number of answer options (up to 26), local examination guidelines recommend 5, 6, 7, or 8 answer options. According to local examination guidelines, MTF items might contain 4, 5, or 6 statements.

For Pick-N items, a total of 24 different scoring methods have been described in the literature [6]. Moreover, for MTF items, a large variety of scoring methods exist, and a total of 27 scoring methods have been described in the literature [5]. By removing duplicate scoring algorithms, 41 scoring algorithms were identified and were retrospectively applied to examinees’ markings of both multiple-select multiple-choice item types.

### Electronic Examinations

Prior to their use, all items were subjected to a review process at the department responsible for the respective examination. During electronic examinations, answer options/statements were displayed and permuted for each examinee using UCAN’s CAMPUS Examination software [17]. Until the end of the examination, examinees were able to modify their markings. Total examination time was calculated based on 90 seconds per item.

For Pick-N items, examinees had to mark only the true answer options (*t*). For each item, the number of true answer options was displayed to the examinees. Marking more answer options as true than the given number of *t* was technically impossible. If examinees marked fewer answer options than *t* as true, a warning message was shown indicating that they were intended to select *t* answer options. Despite the warning message, examinees were allowed to continue without selecting *t* answer options. Within the context of MTF items, examinees were required to mark each statement as either true or false, and there was no possibility to omit individual statements.

For all examinations (usually consisting of 20 to 30 items), a uniform pass mark of 60% (ie, 0.6 credit points) was used irrespective of the included item types according to local examination guidelines.

### Examination Data

Written examinations of the Department of Preventive Dentistry, Periodontology and Cariology and the Department of Prosthodontics of the undergraduate dental curriculum (1st to 10th semester) at the University Medical Center Göttingen were retrospectively screened for multiple-select multiple-choice items. Due to the overall lower number of Pick-N items, Pick-N items and examination data were retrieved from all examinations with at least five participants between 2016 and 2020. In case of Pick-N items used in multiple examinations, only the version and marking events from the examination with the most examinees or the first examination (in cases of the same number of examinees) were assessed. MTF items and corresponding examination data were retrieved from a previous publication [18] containing items from examinations with at least five participants during winter term 2016/2017 only. If MTF items were used in multiple eligible examinations, marking events from all examinations were combined. To allow for comparison, MTF items from the previous publication were limited to the fields of Operative Dentistry and Prosthodontics.

### Quality Criteria of Items

Judgement regarding the use of an appropriate item type was based on the definition by Krebs [10]. In order to further evaluate the quality of identified items, a validated checklist regarding formal quality criteria, presence of cues, and content correctness was used (Table 1) [18]. Formal quality and presence of cues were jointly assessed by 3 authors (PK, MH, and TR) to classify items for the subsequent analyses. Content validity was assessed by 2 expert clinicians (AW for items within the field of Operative Dentistry; TW for items regarding Prosthodontics).

**Table 1:** Checklist for the quality assessment of items as described previously [18].

Quality parameter			Items fulfilling the criteria				
			Pick-N (N=48), n (%)		Multiple-True-False (N=18), n (%)		
**Formal**							
	Is the item linguistically correct?	25 (52)		12 (67)			
	Are the answer options homogeneous (eg, no double negatives, approximately equal length of statements)?	40 (83)		11 (61)			
	Are students of the subject able to understand the question?	46 (96)		15 (83)			
	Is the correct item type used?	18 (38)		14 (78)			
**Cues**							
	Have cues (eg, grammar hints, correct statement is the longest option, diametrical statements, statements which mutually exclude/condition each other, verbal association between question and statements, absolute formulations such as never or always) been avoided?	27 (56)		9 (50)			
**Content**							
	Is the content correct?	44 (92)		18 (100)			
	Are answer options homogeneous regarding their content?	47 (98)		13 (72)			

### Statistical Analysis

Scoring results for all marking events (ie, individual student entries on a single item) of identified Pick-N and MTF items were calculated according to the identified scoring algorithms shown in Multimedia Appendix 1, using Excel for Mac (version 16.39; Microsoft Corp). Based on these results, a mean score across all examinees and items was calculated for each scoring algorithm and item type. Separately for Pick-N and MTF items, differences between the mean scores of all scoring methods were assessed by the Kruskal-Wallis test.

The effect of item quality (use of an appropriate item type [yes vs no] and absence of cues [yes vs no]) on mean raw scores was assessed by the Wilcoxon rank-sum test. Raw scores were derived from method 10 (Partial Scoring 1/*n*, PS_1/_*_n_*), which awards partial credit equally for each correctly marked answer option/statement.

Separately for each scoring method, the likelihood of achieving a score of ≥0.6 was assessed by multiple logistic regression analyses. The use of an inappropriate item type (yes vs no) and presence of cues (yes vs no) were simultaneously entered as predictor variables. A dichotomous outcome was defined as a score at or above pass mark (≥0.6 credit points) versus below pass mark (<0.6 credit points).

All calculations were performed using the software R [19] (version 4.0.4) and the package “PMCMR” (version 4.3). The level of significance was set at α=.05.

## Results

### Marking Events

A total of 48 Pick-N and 18 MTF items were included. Items presented 5, 6, or 7 answer options (Pick-N), or 5 or 6 statements (MTF). A total of 1931 (Pick-N) and 828 (MTF) marking events were investigated. On average, for Pick-N and MTF items, each item was answered by 40.2 (SD 5.7) and 46.0 (SD 30.7) examinees.

### Scoring Results

Except for method 9 (Monash Medical School Scheme), which has only been described for cases of *n*=4, all identified scoring methods were applied on all included items.

For both item types, mean scores differed significantly between scoring methods (*P*<.001). For Pick-N items, mean scores per item varied between 0.5, when applying method 16 (Guessing Penalty), and 0.98, when applying method 2 (Dichotomized MTF) or method 32 (Formula 3 by Blasberg et al [8]). Overall, mean scores of ≥0.90 per item were achieved when using method 2 (Dichotomized MTF), method 32 (Formula 3 by Blasberg et al [8]), method 15 (Guessing Fair Penalty), or method 29 (Formula 6 by Duncan and Milton [20]). For MTF items, mean scores per item varied between 0.02, when applying method 16 (Guessing Penalty), and 0.96, when applying method 2 (Dichotomized MTF). Only 2 scoring methods resulted in mean scores of ≥0.90 (method 2 [Dichotomized MTF] and method 15 [Guessing Fair Penalty]). The results of further scoring methods are shown in Table 2.

**Figure 2:**
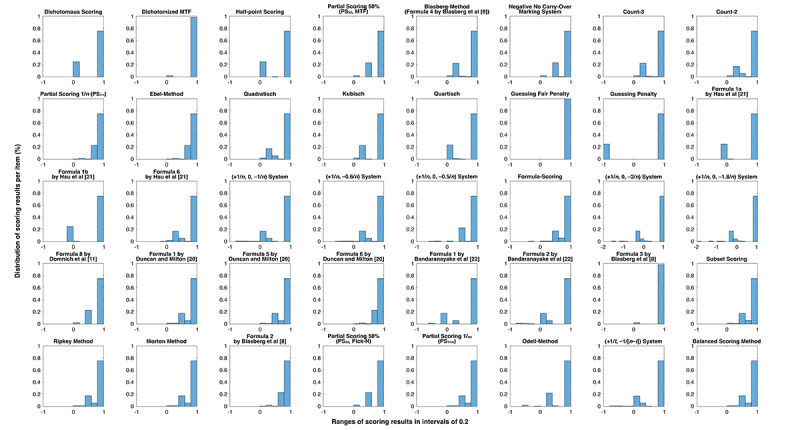
Distribution of scoring results per item among all 1931 marking events of Pick-N items. The ranges of scoring results are shown on the x-axis in intervals of 0.2 with a scale ranging from −2 or −1 to +1 credit points per item. MTF: Multiple-True-False.

**Figure 3:**
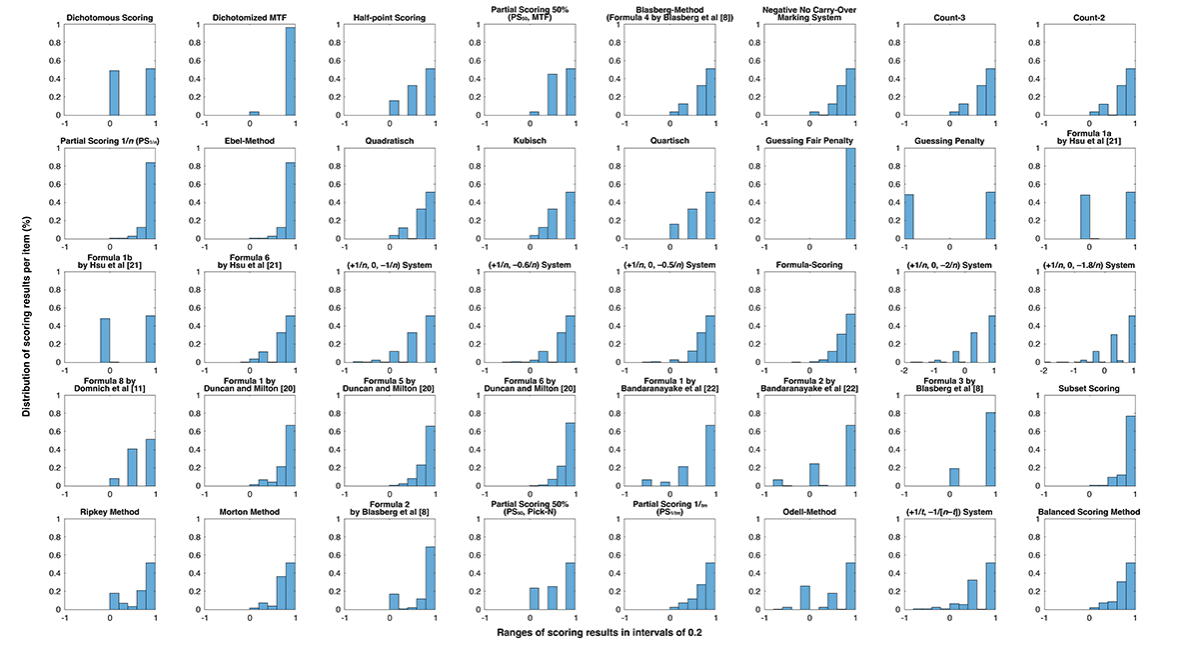
Distribution of scoring results per item among all 828 marking events of Multiple-True-False (MTF) items. The ranges of scoring results are shown on the x-axis in intervals of 0.2 with a scale ranging from −2 or −1 to +1 credit points per item.

For Pick-N and MTF item types, histograms showing the distribution of scoring results from different scoring methods are presented in Figures 2 and 3, respectively. As depicted, different scoring methods allow for different levels of partial credit.

**Table 2:** Mean scoring results across all examinees per item for different scoring methods.

Method number	Scoring method	Scoring result, mean (SD)	
		Pick-N	Multiple-True-False (MTF)
1	Dichotomous Scoring	0.752 (0.432)	0.512 (0.500)
2	Dichotomized MTF	0.982 (0.133)	0.963 (0.190)
3	Half-point Scoring	0.752 (0.431)	0.675 (0.371)
4	Partial Scoring 50% (PS_50_, MTF)^a^	0.867 (0.241)	0.737 (0.285)
5	Blasberg-Method (Formula 4 by Blasberg et al [8])	0.807 (0.340)	0.734 (0.312)
6	Negative No Carry-Over Marking System	0.851 (0.267)	0.794 (0.251)
7	Count-3	0.830 (0.299)	0.771 (0.227)
8	Count-2	0.838 (0.288)	0.773 (0.275)
9	Monash Medical School Scheme^b^	N/A^c^	N/A
10	Partial Scoring 1/*n* (PS_1/__*n*_)	0.899 (0.183)	0.861 (0.173)
11	Ebel-Method	0.899 (0.183)	0.861 (0.173)
12	Quadratisch	0.842 (0.279)	0.772 (0.262)
13	Kubisch	0.808 (0.337)	0.709 (0.319)
14	Quartisch	0.787 (0.373)	0.664 (0.359)
15	Guessing Fair Penalty	0.953 (0.083)	0.903 (0.099)
16	Guessing Penalty	0.504 (0.864)	0.024 (1.000)
17	Formula 1a by Hsu et al [21]	0.742 (0.449)	0.493 (0.520)
18	Formula 1b by Hsu et al [21]	0.744 (0.446)	0.496 (0.517)
19	Formula 6 by Hsu et al [21]	0.829 (0.303)	0.762 (0.282)
20	(+1/*n*, 0, –1/*n*) System	0.798 (0.366)	0.723 (0.347)
21	(+1/*n*, –0.6/*n*) System	0.839 (0.292)	0.778 (0.277)
22	(+1/*n*, 0, –0.5/*n*) System	0.849 (0.274)	0.792 (0.260)
23	Formula-Scoring	0.875 (0.226)	0.827 (0.216)
24	(+1/*n*, 0, –2/*n*) System	0.697 (0.548)	0.584 (0.520)
25	(+1/*n*, 0, –1.8/*n*) System	0.718 (0.512)	0.612 (0.485)
26	Formula 8 by Domnich et al [11]	0.866 (0.243)	0.716 (0.319)
27	Formula 1 by Duncan and Milton [20]	0.879 (0.222)	0.851 (0.234)
28	Formula 5 by Duncan and Milton [20]	0.893 (0.194)	0.856 (0.187)
29	Formula 6 by Duncan and Milton [20]	0.904 (0.174)	0.868 (0.170)
30	Formula 1 by Bandaranayake et al [22]	0.757 (0.443)	0.702 (0.468)
31	Formula 2 by Bandaranayake et al [22]	0.790 (0.381)	0.652 (0.544)
32	Formula 3 by Blasberg et al [8]	0.982 (0.133)	0.808 (0.394)
33	Subset Scoring	0.896 (0.189)	0.868 (0.170)
34	Ripkey Method	0.879 (0.222)	0.692 (0.378)
35	Morton Method	0.879 (0.222)	0.802 (0.233)
36	Formula 2 by Blasberg et al [8]	0.899 (0.183)	0.735 (0.364)
37	Partial Scoring 50% (PS_50_, Pick-N)^a^	0.866 (0.243)	0.638 (0.410)
38	Partial Scoring 1/*t*_*m*_ (PS_1/__*tm*_)	0.879 (0.222)	0.778 (0.258)
39	Odell-Method	0.824 (0.319)	0.595 (0.471)
40	(+1/*t*, –1/[*n*–*t*]) System	0.791 (0.378)	0.737 (0.339)
41	Balanced Scoring Method	0.879 (0.222)	0.785 (0.249)

^a^Within the context of Pick-N and Multiple-True-False items, the scoring method named Partial Scoring 50% (PS_50_) is related to different scoring methods.

^b^Only used in case of 4 answer options/statements per item.

^c^N/A: not applicable.

### Impact of Item Quality on Scoring Results

A total of 30 (63%) Pick-N items should have been used as MTF items, while 4 (22%) MTF items should have been used as Pick-N items instead. Presence of at least one cue was found in 21 out of 48 (44%) Pick-N items, while at least one cue was identified in 9 out of 18 (50%) MTF items. However, the content of items was formally correct in 44 out of 48 (92%) Pick-N items and all (100%) MTF items.

Inappropriately used Pick-N items (ie, these items should have been written as MTF items instead) resulted in lower mean raw scores (mean 0.88, SD 0.20 vs mean 0.93, SD 0.16; *P*<.001), while inappropriately used MTF items resulted in higher mean raw scores (mean 0.88, SD 0.19 vs mean 0.85, SD 0.17; *P*=.001). Mean raw scores from items with and without cues differed for MTF items (mean 0.91, SD 0.15 vs mean 0.84, SD 0.18; *P*<.001), but not for Pick-N items (mean 0.89, SD 0.18 vs mean 0.90, SD 0.18; *P*=.09).

For Pick-N items used inappropriately, most scoring methods showed a lower likelihood of achieving a score of ≥0.6 compared to credit from proper Pick-N items (odds ratio [OR] ≤0.559; Table 3). For items written up inappropriately in MTF style, most scoring methods showed a greater likelihood of achieving a score of ≥0.6 compared to items that were designed appropriately (Table 3). The highest effect was found for method 38 (Partial Scoring 1/*t_m_*, PS_1/_*_tm_*; OR 5.724) and method 27 (Formula 1 by Duncan and Milton [20]; OR 4.776). Only 2 scoring methods showed a lower proportion of scores ≥0.6 when an inappropriate item type was used (method 32 and method 36 [Formula 2 and 3 by Blasberg et al [8]], both OR 0.625).

Within Pick-N items, the presence of cues was associated with a greater likelihood of achieving a score of ≥0.6 (equaling scores at or above the pass mark that is ≥60% of the total score) for a minority of scoring methods only (Table 3). Differences in the likelihood of scores ≥0.6 between items with and without cues were most pronounced when using methods 27, 34, 35, 38, and 41 (all OR 1.394). No scoring method resulted in a lower proportion of scores ≥0.6 in case of cues being present. Different results were found for MTF items. For most scoring methods, the presence of cues was associated with a greater likelihood of achieving a score of ≥0.6 (Table 3). Scoring methods 30 and 31 (Formula 1 and 2 by Bandaranayake et al. [22]) showed the highest susceptibility to cues (both OR 6.977). Only 2 scoring methods showed a lower proportion of scores ≥0.6 in the presence of cues (methods 32 and 36 [Formula 2 and 3 by Blasberg et al [8]], both OR 0.451).

**Table 3:** Results of multiple logistic regression analyses regarding the effect of item quality on scoring results (≥0.6 vs <0.6 credit points).

Method number	Pick-N						Multiple-True-False					
	Use of inappropriate item type (yes vs no)			Presence of cues (yes vs no)			Use of inappropriate item type (yes vs no)			Presence of cues (yes vs no)		
	OR^a^ (95% CI)	*P* value	OR (95% CI)		*P* value	OR (95% CI)		*P* value	OR (95% CI)		*P* value	
1	0.553 (0.440-0.693)	<.001	0.882 (0.711-1.094)		.25	1.778 (1.273-2.496)		.001	2.296 (1.707-3.100)		<.001	
2	0.250 (0.092-0.574)	.002	1.887 (0.938-3.982)		.08	0.983 (0.446-2.393)		.97	2.365 (1.015-6.460)		.06	
3	0.553 (0.440-0.693)	<.001	0.882 (0.711-1.094)		.25	1.778 (1.273-2.496)		.001	2.296 (1.707-3.100)		<.001	
4	0.553 (0.440-0.693)	<.001	0.882 (0.711-1.094)		.25	1.778 (1.273-2.496)		.001	2.296 (1.707-3.100)		<.001	
5	0.559 (0.445-0.701)	<.001	0.870 (0.702-1.080)		.21	2.103 (1.300-3.538)		.003	5.432 (3.231-9.730)		<.001	
6	0.559 (0.445-0.701)	<.001	0.870 (0.702-1.080)		.21	2.103 (1.300-3.538)		.003	5.432 (3.231-9.730)		<.001	
7	0.559 (0.445-0.701)	<.001	0.870 (0.702-1.080)		.21	2.103 (1.300-3.538)		.003	5.432 (3.231-9.730)		<.001	
8	0.559 (0.445-0.701)	<.001	0.870 (0.702-1.080)		.21	2.103 (1.300-3.538)		.003	5.432 (3.231-9.730)		<.001	
9	N/A^b^	N/A	N/A		N/A	N/A		N/A	N/A		N/A	
10	0.250 (0.092-0.574)	.002	1.887 (0.938-3.982)		.08	0.983 (0.446-2.393)		.97	2.365 (1.015-6.460)		.06	
11	0.250 (0.092-0.574)	.002	1.887 (0.938-3.982)		.08	0.983 (0.446-2.393)		.97	2.365 (1.015-6.460)		.06	
12	0.559 (0.445-0.701)	<.001	0.870 (0.702-1.080)		.21	2.103 (1.300-3.538)		.003	5.432 (3.231-9.730)		<.001	
13	0.553 (0.440-0.693)	<.001	0.882 (0.711-1.094)		.25	1.778 (1.273-2.496)		.001	2.296 (1.707-3.100)		<.001	
14	0.553 (0.440-0.693)	<.001	0.882 (0.711-1.094)		.25	1.778 (1.273-2.496)		.001	2.296 (1.707-3.100)		<.001	
15	N/A	>.99	N/A		>.99	N/A		>.99	N/A		>.99	
16	0.553 (0.440-0.693)	<.001	0.882 (0.711-1.094)		.25	1.778 (1.273-2.496)		.001	2.296 (1.707-3.100)		<.001	
17	0.553 (0.440-0.693)	<.001	0.882 (0.711-1.094)		.25	1.778 (1.273-2.496)		.001	2.296 (1.707-3.100)		<.001	
18	0.553 (0.440-0.693)	<.001	0.882 (0.711-1.094)		.25	1.778 (1.273-2.496)		.001	2.296 (1.707-3.100)		<.001	
19	0.559 (0.445-0.701)	<.001	0.870 (0.702-1.080)		.21	2.103 (1.300-3.538)		.003	5.432 (3.231-9.730)		<.001	
20	0.559 (0.445-0.701)	<.001	0.870 (0.702-1.080)		.21	2.103 (1.300-3.538)		.003	5.432 (3.231-9.730)		<.001	
21	0.559 (0.445-0.701)	<.001	0.870 (0.702-1.080)		.21	2.103 (1.300-3.538)		.003	5.432 (3.231-9.730)		<.001	
22	0.559 (0.445-0.701)	<.001	0.870 (0.702-1.080)		.21	2.103 (1.300-3.538)		.003	5.432 (3.231-9.730)		<.001	
23	0.489 (0.379-0.629)	<.001	1.364 (1.074-1.737)		.01	2.482 (1.501-4.310)		.001	5.349 (3.178-9.590)		<.001	
24	0.553 (0.440-0.693)	<.001	0.882 (0.711-1.094)		.25	1.778 (1.273-2.496)		.001	2.296 (1.707-3.100)		<.001	
25	0.553 (0.440-0.693)	<.001	0.882 (0.711-1.094)		.25	1.778 (1.273-2.496)		.001	2.296 (1.707-3.100)		<.001	
26	0.553 (0.440-0.693)	<.001	0.882 (0.711-1.094)		.25	1.778 (1.273-2.496)		.001	2.296 (1.707-3.100)		<.001	
27	0.480 (0.371-0.616)	<.001	1.394 (1.098-1.775)		.007	4.776 (2.401-10.911)		<.001	3.799 (2.227-6.877)		<.001	
28	0.486 (0.376-0.625)	<.001	1.374 (1.082-1.750)		.009	3.802 (1.957-8.320)		<.001	6.537 (3.393-14.220)		<.001	
29	0.250 (0.092-0.574)	.002	1.887 (0.938-3.982)		.08	0.927 (0.544-1.635)		.79	2.776 (1.540-5.382)		.001	
30	0.553 (0.440-0.693)	<.001	0.882 (0.711-1.094)		.25	2.993 (2.027-4.494)		<.001	6.977 (4.743-10.515)		<.001	
31	0.553 (0.440-0.693)	<.001	0.882 (0.711-1.094)		.25	2.993 (2.027-4.494)		<.001	6.977 (4.743-10.515)		<.001	
32	0.250 (0.092-0.574)	.002	1.887 (0.938-3.982)		.08	0.625 (0.420-0.940)		.02	0.451 (0.314-0.644)		<.001	
33	0.489 (0.379-0.629)	<.001	1.364 (1.074-1.737)		.01	1.331 (0.788-2.346)		.30	3.679 (2.065-7.074)		<.001	
34	0.480 (0.371-0.616)	<.001	1.394 (1.098-1.775)		.007	1.271 (0.878-1.867)		.21	0.849 (0.618-1.169)		.31	
35	0.480 (0.371-0.616)	<.001	1.394 (1.098-1.775)		.007	4.335 (2.247-9.441)		<.001	3.427 (2.054-6.020)		<.001	
36	0.250 (0.092-0.574)	.002	1.887 (0.938-3.982)		.08	0.625 (0.420-0.940)		.02	0.451 (0.314-0.644)		<.001	
37	0.553 (0.440-0.693)	<.001	0.882 (0.711-1.094)		.25	1.778 (1.273-2.496)		.001	2.296 (1.707-3.100)		<.001	
38	0.480 (0.371-0.616)	<.001	1.394 (1.098-1.775)		.007	5.724 (3.167-11.441)		<.001	0.869 (0.614-1.235)		.43	
39	0.559 (0.445-0.701)	<.001	0.870 (0.702-1.080)		.21	1.864 (1.331-2.626)		<.001	2.395 (1.777-3.244)		<.001	
40	0.553 (0.440-0.693)	<.001	0.882 (0.711-1.094)		.25	0.964 (0.662-1.421)		.85	2.485 (1.704-3.694)		<.001	
41	0.480 (0.371-0.616)	<.001	1.394 (1.098-1.775)		.007	0.984 (0.655-1.504)		.94	1.975 (1.325-3.006)		.001	

^a^OR: odds ratio.

^b^N/A: not applicable.

## Discussion

### Principal Findings

When retrospectively applying the described scoring methods on examination items, the applied scoring method, presence of cues, and use of an inappropriate item type impacted the credit assignment. Therefore, both null hypotheses must be rejected.

Averaged scores differed significantly between different scoring methods for both item types. For Pick-N items, mean scores ranged from 0.50 (method 16) to 0.98 (method 2) credit points for the same markings, while MTF items showed an even bigger range of 0.02 (method 16) to 0.96 (method 2) credit points. Both the use of an inappropriate item type and presence of cues significantly impacted the scoring results. Inappropriately used Pick-N items resulted in lower mean raw scores (mean 0.88, SD 0.20 vs mean 0.93, SD 0.16), while inappropriately used MTF items resulted in higher mean raw scores (mean 0.88, SD 0.19 vs mean 0.85, SD 0.17). The mean raw score from MTF items with cues was 0.91 (SD 0.15), while items without cues resulted in a lower mean raw score of 0.84 (SD 0.18). These differences emphasize the effects of different scoring methods, presence of cues, and inappropriately used item types, as examinees might either pass or fail the examination based on an assumed fixed pass mark of 60% (ie, 0.6 credit points on average). For most scoring methods, item quality impacted the likelihood of scores ≥0.6. Inappropriately used Pick-N items showed a lower likelihood of scores ≥0.6, while inappropriately used MTF items showed a higher likelihood of scores ≥0.6. MTF items containing at least one cue showed a higher likelihood of scores ≥0.6 than items without cues.

Two different types of multiple-select multiple-choice items were used in this study. Between Pick-N and MTF items, examinees’ decision-making and response behaviors are fundamentally different. In Pick-N items, the number of true answer options to be selected is disclosed to examinees. Therefore, marking answer options within Pick-N items is dependent on the marking of all other answer options within the same item [6]. The metric *expected chance score* [23] from random guessing amounts to 
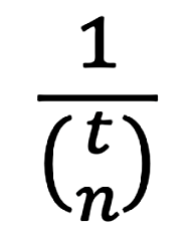
. In contrast, every statement within an MTF item might be either true or false (including zero or even all statements). Thereby, examinees are forced to independently assess each statement as true or false, and the expected chance score amounts to 0.5*^n^* [5]. Based on these theoretical implications, lower mean scores can be expected if examinees are not aware of the total number of correct answer options/statements (such as in MTF items). To address these differences regarding the relative item difficulty between both item types, local examination guidelines might suggest different scoring methods or pass marks for both item types. This study found scores resulting from both Pick-N and MTF items to vary based on the selected scoring methods. Therefore, examination results should only be interpreted in light of the employed scoring method or methods.

Within this study, items were extracted from different examinations covering a broad range of topics and learning objectives. Therefore, no direct comparison of the item difficulty between MTF and Pick-N items was made. Instead, the effect of item quality was assessed. Inappropriately used MTF items resulted in higher mean raw scores, while inappropriately used Pick-N items resulted in lower mean raw scores. This observation might be attributed to the definitions regarding the correct use of Pick-N and MTF items. MTF items require more complex statements than Pick-N items [7,10]. As a result, MTF items are likely to be overall more complex, requiring higher cognitive skills from examinees. If local examination guidelines suggest different scoring methods or pass marks for both item types to overcome the above-mentioned differences between both item types, the use of an inappropriate item type might result in either an inflation (in case of inappropriately used MTF items) or deduction (in case of inappropriately used Pick-N items) of scores at or above the pass mark.

Besides item types used inappropriately, cues were found to impact scoring results. While the mean raw scores of Pick-N items with and without cues did not differ, the presence of cues in MTF items resulted in a higher proportion of correctly marked statements. Thus, MTF showed a higher susceptibility to cues. As examinees are likely to consider cues during their decision-making process, educators should carefully evaluate each item using a checklist for quality assessment and cues (eg, grammar hints, diametrical statements, or absolute formulations) to eliminate cues prior to its use in an examination.

Besides selecting an appropriate item type, educators need to select an adequate scoring method. In contrast to single-choice items, scoring of multiple-select items is complicated as examinees might give partially correct responses. In recent systematic reviews, a total of 41 scoring methods for MTF and Pick-N items were described [5,6]. Scoring methods focusing on the number of correct responses instead of the number of true answer options/statements marked as true (*t_m_*) and accurately discriminating between different levels of knowledge are most frequently recommended [5]. Scoring methods yielding negative scores should not be used because of jurisdictional reasons [5,18,24]. However, available item types and scoring methods are often set by local examination guidelines.

Overall, the results of this retrospective assessment of real examination data confirm the assumption that credit assignment on MTF and Pick-N items differs between varying scoring methods. Furthermore, it was shown that item quality characteristics like selection of an appropriate item type and avoidance of cues have a significant effect on scoring results in the case of most scoring methods.

### Strengths and Limitations

The strengths of this assessment include the use of up to 41 scoring methods and a high number of marking events (Pick-N items: 1931; MTF items: 828). Previous studies on this topic were based on theoretical calculations only [5,6] or used a smaller number of different scoring methods/item types [18]. For each item, quality was assessed based on a validated checklist. However, a number of limitations are present. First, items were derived from previous examinations, which resulted in an unequal distribution of both item types. While 48 Pick-N items were included, only 18 MTF items were assessed. Second, all items were extracted from different examinations covering a broad range of topics. Therefore, no direct comparison of the item difficulty between MTF and Pick-N items was possible. Third, no further predictor variables (eg, student-related variables such as age and gender) were available due to the retrospective and anonymous design.

### Future Directions

To address these limitations, further prospective studies should evaluate different scoring methods and item types by employing matched items on the same learning objectives. Moreover, further predictor variables (eg, student-related variables such as age and gender) should be considered.

### Conclusion

Educators should pay attention when using multiple-select multiple-choice items. Scoring and awarding credit are more complex for multiple-select multiple-choice items than for single-choice items. This manuscript may guide educators to make informed decisions regarding the use of multiple-select multiple-choice items.

Different item types, different scoring methods, and presence of cues are likely to impact examinees’ scores and overall examination results. Therefore, educators should carefully select the most appropriate item type. Moreover, cues should be avoided as far as possible. Finally, examination results should be interpreted in light of the used item type and applied scoring method.

## Appendix

Multimedia Appendix 1 Scoring methods for Pick-N and Multiple-True-False items as described in the literature.

## Abbreviations

MTF: Multiple-True-False
OR: odds ratio

## Data Availability

The data sets generated during or analyzed during this study are available from the corresponding author on reasonable request.
