# Supplementary material for: Use of Multiple-Select Multiple-Choice Items in a Dental Undergraduate Curriculum: Retrospective Study Involving the Application of Different Scoring Methods
Source: JMIR Med Educ. 2023 Mar 27;9:e43792. doi: 10.2196/43792 (PMC10131704; doi:10.2196/43792)
Supplement: Multimedia Appendix 1 [file mededu_v9i1e43792_app1.docx]

**Multimedia Appendix 1**

| **Method number** | **Focus** | **Scoring range** | **Scoring method** | **Item type** | | **Scoring algorithm** |
| --- | --- | --- | --- | --- | --- | --- |
|  |  |  |  | Pick-N (N=24) | Multiple-True-False (N=27) |  |
| 1 | Examinees correct responses (*i*) | 0 to 1 | Dichotomous Scoring | x | x | $f=\left\{ \begin{aligned} 1 if i=n \\ \\ 0 otherwise \end{aligned} \right.$ |
| 2 |  |  | Dichotomized MTF |  | x | $f=\left\{ \begin{aligned} 1 if i>\frac{n}{2} \\ \\ 0 otherwise \end{aligned} \right.$ |
| 3 |  |  | Half-point Scoring |  | x | $f=\left\{ \begin{aligned} 1 if i=n \\ 0.5 if i=n-1 \\ 0 otherwise \end{aligned} \right.$ |
| 4 |  |  | Partial Scoring 50% (PS_50_, MTF)^a^ |  | x | $f=\left\{ \begin{aligned} 1 if i=n \\ 0.5 if \frac{n}{2}<i<n \\ 0 otherwise \end{aligned} \right.$ |
| 5 |  |  | Blasberg-Method (Formula 4 by Blasberg et al [8]) | x | x | Pick-N: $f=\left\{ \begin{aligned} \frac{i}{n}-\frac{n-i}{n}=\frac{2i-n}{n} if i>\frac{n}{2} \\ \\ 0 otherwise \end{aligned} \right.$  MTF: $f=\left\{ \begin{aligned} \frac{i}{n}-\frac{n-i-o}{n}=\frac{2i-n+o}{n} if i>\frac{n}{2} \\ \\ 0 otherwise \end{aligned} \right.$ |
| 6 |  |  | Negative No Carry-Over Marking System |  | x | $f=\left\{ \begin{aligned} \frac{i}{n}-0.5\frac{n-i-o}{n}=\frac{1.5i-0.5n+0.5o}{n} if i>\frac{n}{3} \\ \\ 0 otherwise \end{aligned} \right.$ |
| 7 |  |  | Count-3 | x | x | $f=\left\{ \begin{aligned} \frac{i-\left\lfloor\frac{n}{2} \right\rfloor}{n-\left\lfloor\frac{n}{2} \right\rfloor} if i>\frac{n}{2} \\ \\ 0 otherwise \end{aligned} \right.$ |
| 8 |  |  | Count-2 | x | x | $f=\left\{ \begin{aligned} \frac{i-\left\lceil\frac{n}{2} \right\rceil+1}{n-\left\lceil\frac{n}{2} \right\rceil+1} if i\geq\frac{n}{2} \\ \\ 0 otherwise \end{aligned} \right.$ |
| 9 |  |  | Monash Medical School Scheme^b^ |  | x | $f=\left\{ \begin{aligned} {1 if i=4 \atop0.6 if i=3} \\ 0.2 if i=2 \\ 0 otherwise \end{aligned} \right.$ |
| 10 |  |  | Partial Scoring 1/*n* (PS_1/_*_n_*) | x | x | $f=\frac{i}{n}$ |
| 11 |  |  | Ebel-Method |  | x | $f=\frac{i}{n}+\frac{o}{n^{2}}$ |
| 12 |  |  | Quadratisch |  | x | $f=\left( \frac{i}{n} \right)^{2}$ |
| 13 |  |  | Kubisch |  | x | $f=\left( \frac{i}{n} \right)^{3}$ |
| 14 |  |  | Quartisch |  | x | $f=\left( \frac{i}{n} \right)^{4}$ |
| 15 |  |  | Guessing Fair Penalty |  | x | $f=\left\{ \begin{aligned} 1 if i=n \\ \\ 1-\frac{1}{n} otherwise \end{aligned} \right.$ |
| 16 |  | ≥–1 to 1 | Guessing Penalty |  | x | $f=\left\{ \begin{aligned} 1 if i=n \\ \\ -1 otherwise \end{aligned} \right.$ |
| 17 |  |  | Formula 1a by Hsu et al [21] | x |  | $f=\left\{ \begin{aligned} 1 if i=n \\ 0 if m_{t}=0 \\ - \frac{1}{25} otherwise \end{aligned} \right.$ |
| 18 |  |  | Formula 1b by Hsu et al [21] | x |  | $f=\left\{ \begin{aligned} 1 if i=n \\ 0 if m_{t}=0 \\ - \frac{1}{30} otherwise \end{aligned} \right.$ |
| 19 |  |  | Formula 6 by Hsu et al [21] | x |  | $f=\left\{ \begin{aligned} 1 if i=n \\ 0 if m_{t}=0 \\ \frac{i}{n}-\frac{\binom{n}{n-i}}{2^{n}} otherwise \end{aligned} \right.$ |
| 20 |  |  | (+1/*n*, 0, –1/*n*) System | x | x | Pick-N: $f=\left\{ \begin{aligned} 0 if m_{t}=0 \\ \\ \frac{i}{n}-\frac{n-i}{n}=\frac{2i-n}{n} otherwise \end{aligned} \right.$  MTF: $f=\frac{i}{n}-\frac{n-i-o}{n}=\frac{2i-n+o}{n}$ |
| 21 |  |  | (+1/*n*, –0.6/*n*) System |  | x | $f=\frac{i}{n}-0.6\frac{n-i}{n}=\frac{1.6i-0.6n}{n}$ |
| 22 |  |  | (+1/*n*, 0, –0.5/*n*) System |  | x | $f=\frac{i}{n}-0.5\frac{n-i-o}{n}=\frac{1.5i-0.5n+0.5o}{n}$ |
| 23 |  |  | Formula-Scoring | x | x | $f=\frac{i}{n}-\frac{n-i}{n (n-1)}=\frac{i-1}{n-1}$ |
| 24 |  | ≥–2 to 1 | (+1/*n*, 0, –2/*n*) System |  | x | $f=\frac{i}{n}-2\frac{n-i-o}{n}=\frac{3i-2n+2o}{n}$ |
| 25 |  |  | (+1/*n*, 0, –1.8/*n*) System |  | x | $f=\frac{i}{n}-1.8\frac{n-i-o}{n}=\frac{2.8i-1.8n+1.8o}{n}$ |
| 26 | True answer options / statements identified as true (*t_m_*) | 0 to 1 | Formula 8 by Domnich et al [11] | x |  | $f=\left\{ \begin{aligned} 1 if i=n \\ 0.5 if \frac{t}{2}\leq t_{m}<t \\ 0 otherwise \end{aligned} \right.$ |
| 27 |  |  | Formula 1 by Duncan and Milton [20] | x |  | $f=\frac{t_{m}}{t}$ |
| 28 |  |  | Formula 5 by Duncan and Milton [20] | x |  | $f=2\frac{t_{m}}{n+t}+\frac{i-t_{m}}{n+t}=\frac{t_{m}+i}{n+t}$ |
| 29 |  |  | Formula 6 by Duncan and Milton [20] | x |  | $f=\frac{t_{m}}{n+(n-t)}+2\frac{i-t_{m}}{n+(n-t)}=\frac{2 i{-t}_{m}}{2 n-t}$ |
| 30 |  | ≥–1 to 1 | Formula 1 by Bandaranayake et al [22] |  | x | $f=\left\{ \begin{aligned} 1 if m_{t}=t=0 \\ \\ \frac{t_{m}-(t-t_{m})}{t}=\frac{2t_{m}-t}{t} otherwise \end{aligned} \right.$ |
| 31 |  |  | Formula 2 by Bandaranayake et al [22] |  | x | $f=\left\{ \begin{aligned} \frac{t_{m}}{t} if n=t \\ 1 if m_{t}=t=0 \\ \frac{t_{m}}{t}-\frac{t-t_{m}}{n-t}=\frac{{n t}_{m}-t^{2}}{t (n-t)} otherwise \end{aligned} \right.$ |
| 32 | True answer options/ statements identified as true (*t_m_*), credit is reduced if *m_t_ > t* | 0 to 1 | Formula 3 by Blasberg et al [8] | x |  | $f=\left\{ \begin{aligned} 1 if i>\frac{n}{2} and m_{t}\leq t \\ \\ 0 otherwise \end{aligned} \right.$ |
| 33 |  |  | Subset Scoring | x |  | $f=\frac{\left( n-t \right)t_{m}+t(n-t)}{2t(n-t)}-\frac{t\left( m_{t}-t_{m} \right)}{2t\left( n-t \right)}=\frac{n t+n t_{m}-tm_{t}-t^{2}}{2t (n-t)}$ |
| 34 |  |  | Ripkey Method | x | x | Pick-N: $f=\left\{ \begin{aligned} \frac{t_{m}}{t} if m_{t}\leq t \\ \\ 0 otherwise \end{aligned} \right.$  MTF: $f=\left\{ \begin{aligned} \frac{t_{m}}{t} ifm_{t}\leq t and t>0 \\ \\ 1 ifm_{t}=t=0 \\ 0 otherwise \end{aligned} \right.$ |
| 35 |  |  | Morton Method | x |  | $f=\left\{ \begin{aligned} \frac{t_{m}}{t} if m_{t}\leq t \\ \\ \frac{t_{m}}{m_{t}} otherwise \end{aligned} \right.$ |
| 36 |  |  | Formula 2 by Blasberg et al [8] | x |  | $f=\left\{ \begin{aligned} \frac{i}{n} if m_{t}\leq t \\ \\ 0 otherwise \end{aligned} \right.$ |
| 37 |  |  | Partial Scoring 50% (PS_50_, Pick-N)^a^ | x |  | $f=\left\{ \begin{aligned} 1 if i=n \\ 0.5 if \frac{t}{2}\leq t_{m}<t \\ 0.5 if \frac{t_{m}-(m_{t}-t)}{t}{\geq0.5 and m}_{t}>t \\ 0 otherwise \end{aligned} \right.$ |
| 38 |  |  | Partial Scoring 1/*t_m_* (PS_1/_*_tm_*) | x |  | $f=\left\{ \begin{aligned} \\ \frac{t_{m}}{t} if m_{t}\leq t \\ \frac{t_{m}-(m_{t}-t)}{t} otherwise \\ \end{aligned} \right.$ |
| 39 |  | ≥–1 to 1 | Odell-Method | x |  | $f =\left\{ \begin{aligned} \frac{i}{n}-\frac{n-i}{n (\frac{n-t}{t})}=\frac{i-t}{n-t} if m_{t}\leq t \\ \\ 0 otherwise \end{aligned} \right.$ |
| 40 |  |  | (+1/*t*, –1/[*n*–*t*]) System | x | x | Pick-N: $f=\frac{t_{m}}{t}-\frac{m_{t}-t_{m}}{n-t}$  MTF: $f=\left\{ \begin{aligned} \frac{t_{m}}{t} if n=t \\ 1 if m_{t}=t=0 \\ \frac{t_{m}}{t}-\frac{m_{t}-t_{m}}{n-t} otherwise \end{aligned} \right.$ |
| 41 |  |  | Balanced Scoring Method | x | x | Pick-N: $f=\left\{ \begin{aligned} \frac{t_{m}}{t} if m_{t}\leq t \\ \\ \frac{t_{m}}{t}-\frac{m_{t}-t}{n-t} otherwise \end{aligned} \right.$  MTF: $f=\left\{ \begin{aligned} \frac{t_{m}}{t} if m_{t}\leq t and t>0 \\ 1 if m_{t}=t=0 \\ -\frac{m_{t}}{n} if t=0 and m_{t}>0 \\ \frac{t_{m}}{t}-\frac{m_{t}-t}{n-t} otherwise \end{aligned} \right.$ |

Scoring methods for Pick-N and Multiple-True-False (MTF) items as described in literature [5,6]. *f*: resulting score, *i*: number of correct responses by examinee (*n*≥*i*≥0), *m_t_*: overall number of answer options/statements marked as true by examinee, *n*: number of answer options/statements in item, *o*: number of statements omitted by examinee (if possible, otherwise *o*=0), *t*: number of true answer options/statements in item, *t_m_*: number of true answer options/statements marked as true by examinee (*t_m_*≤*t*).

^a^Within the context of Pick-N and Multiple-True-False items, the scoring method named Partial Scoring 50% (PS_50_) is related to different scoring methods.

^b^Only used in case of 4 answer options/statements per item.
